# Patterns of Medicare-funded primary health and specialist consultations in Aboriginal and non-Aboriginal Australians in the two years before hospitalisation for ischaemic heart disease

**DOI:** 10.1186/s12939-018-0826-9

**Published:** 2018-08-02

**Authors:** Tiew-Hwa Katherine Teng, Judith M. Katzenellenbogen, Elizabeth Geelhoed, Anthony S. Gunnell, Matthew Knuiman, Frank M. Sanfilippo, Joseph Hung, Qun Mai, Alistair Vickery, Sandra C. Thompson

**Affiliations:** 1 Western Australian Centre for Rural Health, School of Population and Global Health, The University of Western Australia (M431), 35 Stirling Highway, Perth, WA 6009 Australia; 2 School of Population and Global Health, UWA, Perth, Australia; 3 School of Allied Health, UWA, Perth, Australia; 4 School of Medicine, Sir Charles Gairdner Hospital Unit, UWA, Perth, Australia; 5 Department of Health, Perth, Western Australia Australia; 6 Division of General Practice, School of Medicine, Faculty of Health and Medical Sciences, UWA, Perth, Australia

**Keywords:** Health care disparity, Aboriginal health, Indigenous health services, Ischaemic heart disease, Administrative datasets, Primary health care

## Abstract

**Background:**

Ischaemic heart disease (IHD) remains the leading cause of morbidity and mortality for both Aboriginal and non-Aboriginal Australians. Patterns of primary and specialist care in patients leading up to the first hospitalisation for IHD potentially impact on prevention and subsequent outcomes. We investigated the differences in general practice (GP), specialist and emergency department (ED) consultations, and associated resource use in Aboriginal and non-Aboriginal people in the two years preceding hospitalisation for IHD.

**Methods:**

Linked-data were used to identify first IHD admissions for Western Australians aged 25–74 years in 2002–2007. Person-linked GP, specialist and ED consultations were obtained from the Medicare Benefits Schedule (MBS) and ED records to assess health care access and costs for the preceding 2 years.

**Results:**

Aboriginal people constituted 4.7% of 27,230 IHD patients, 3.5% of 1,348,238 MBS records, and 14% of 33,170 ED presentations. Aboriginal (vs. non-Aboriginal) people were younger (mean 50.2 vs 60.5 years), more commonly women (45.2% vs 28.4%), had more comorbidities [Charlson index≥1, 35.2% vs 26.3%], were more likely to have had GP visits (adjusted rate-ratio 1.07, 95% CI 1.02–1.12), long/prolonged (16.0% vs 11.9%) consults and non-vocationally registered GP consults (17.1% vs 3.2%), but less likely to received specialist consults (mean 1.0 vs 4.1). Mean number of urgent/semi-urgent ED presentations in the year preceding the IHD admission was higher in Aboriginal people (2.9 vs 1.9). Aboriginal people incurred 2.7% of total associated MBS expenditure (estimated at $59.7 million). Mean total cost per person was 43.3% lower in Aboriginal patients, with cost differentials being greatest in diabetic and chronic kidney disease patients.

**Conclusions:**

Despite being over-represented in urgent/semi-urgent ED presentations and admissions for IHD, Aboriginal people were under-resourced compared with the rest of the population, particularly in terms of specialist care prior to first IHD hospitalisation. The findings underscore the need for better primary and specialist shared care delivery models particularly for Aboriginal people.

## Background

Ischaemic heart disease (IHD) remains the leading cause of morbidity and mortality for both Aboriginal and non-Aboriginal populations [1, 2]. The imperative to prevent the first episode of IHD remains strong given that sudden cardiac death occurs on first presentation for one in five [3]. Chronic diseases account for 70% of the gap in total disease burden between Aboriginal and non-Aboriginal Australians [4]. Despite medical advances, cardiovascular disease (CVD) contribute one–fifth of the differential in total disease burden between Aboriginal and non-Aboriginal Australians [4], with greater burden of chronic diseases, such as diabetes and chronic kidney disease (CKD) in the former subpopulation [5].

In Australia, private and public health services exist side by side, with private health insurance (covering part costs of private hospital and specialist services) only accessed by those who can afford to pay. Through Australia’s universal health insurance scheme, public hospital inpatient and outpatient services are free to patients. Primary care services are substantially subsidised through Medicare, both in mainstream services and Aboriginal Medical Services [6], which include additional schemes to better meet Aboriginal health needs [7, 8], e.g. reduced cost of prescribed medicines through Closing the Gap scheme. In Western Australia, medical specialist services are concentrated in Perth, the capital city, with most of the rural population (including 60% of the Aboriginal population) needing to travel considerable distances for access. Rural services have difficulties in attracting health professionals, with workforce retention a further challenge. Despite the fact that Aboriginal Australians have access to primary care through multiple avenues, a recent cost analysis highlighted that 60% of the health expenditure on Aboriginal Australians was on secondary care in public hospitals [9]. However, primary health care (Medicare services and medicines) expenditure per person was significantly less than for non-Aboriginal Australians [9, 10], suggesting underinvestment of funds into prevention, early intervention, secondary prevention and community services for Aboriginal people [10].

General practitioners (GPs) in primary care serve as the gatekeeper to the health system in Australia. GPs have a critical role in the primary prevention of IHD [11], and are ideally situated to provide secondary prevention [11]. The integration between primary, specialist and tertiary care, as a basis of seamless continuity of care is of paramount importance for these patients [11]. GP shortages are however greater in non-metropolitan regions where the majority of Aboriginal people reside [12]. Consequently, policies exist to boost GP numbers in rural areas through financial incentives, and international medical graduates (IMGs) are provided restricted registration to work in areas of unmet need [12]. General practice is recognised as a medical specialty through the General Practice Vocational Register. GPs who have completed their fellowship are vocationally registered (VR GPs) and have access to higher Medicare rebates, while those who are not (Non-VR GPs) utilise lower Medicare rebates except under special programs such as districts of workforce shortage which commonly occurs in disadvantaged and remote areas.

Medicare-rebated (private) specialist services often incur significant co-payment such that patients who experience financial difficulties are under-represented in such practices, relying on hospital out-patient consultations. People who live in rural/remote areas often have limited access to private specialists and are required to travel long distances to access public hospital out-patient services.

Patterns of primary and specialist care in patients leading up to the first admission with IHD potentially impacts on prevention and subsequent outcomes. We aimed to compare the patterns of GP and out-of-hospital specialist consultations, and direct costs of these services in Aboriginal and non-Aboriginal patients in Western Australia (WA) in the 2 years preceding first hospitalisation for IHD. Additionally, we investigated the use of emergency departments (ED) as a substitute for primary health care services.

## Methods

### Study population

A cohort of WA residents aged 25–74 years was identified from administrative hospital data based on first-ever hospitalisation (15-year clearance period) for IHD as a principal discharge diagnosis (‘index hospitalisation’) during the period 2002–2007. IHD was identified from codes I20-I25 of the International Classification of Diseases 10th edition Australian Modification.

### Data sources

Linked state-wide inpatient records from the Hospital Morbidity Data Collection (HMDC) and Emergency Department Data Collection (EDDC) data were obtained from the WA Data Linkage System [13]. National government-held Medicare Benefits Schedule (MBS) data provided details of professional consultations, procedures and diagnostic tests for citizens/permanent residents that universally claim from Medicare. The MBS data do not cover pharmaceutical claims (separate scheme) or outpatient services provided by public hospitals.

### Identification of services and costs

HMDC records were merged with MBS data (2000–2007) to identify the Medicare records for 2 years preceding the index hospitalisation. GP and specialist consultations were identified, and associated costs were compared between Aboriginal and non-Aboriginal patients using the bottom-up itemised MBS costs (scheduled fees). Additional resource costs were estimated by applying costs derived from the MBS for ambulatory community consultations and associated pathology and imaging tests. Person-based costs were aggregated and compared for the two sub-populations. Only direct health care (service provision) costs were examined. The historical consumer price index was used to adjust for changes in costs (2005 as base year).

Similarly, ED presentations in the 2 years preceding the first hospitalisation were identified and relevant data extracted, including demographic and episode fields with presentation time/date and triage scores (≤3 represent immediate/urgent cases; 4 = semi-urgent; 5 = non-urgent) [14].

### Demographic and co-morbidity data

To optimise the estimation of Aboriginal status in routinely-collected data [15, 16], a patient was defined as being Aboriginal if ≥25% of their historical hospital admissions had been coded as Aboriginal. We have used the same definition in previous publications to acknowledge under ascertainment in hospital administrative data collections while avoiding over-inclusion [17, 18]. The Charlson comorbidity index was calculated using the modified Deyo algorithm [19] and individual comorbidities (Table 1) identified using a 5-year look-back period. The Accessibility/Remoteness Index of Australia (ARIA) [20] classifies five categories of residential remoteness and was included as a covariate in the regression analysis. Separately, the greater Perth metropolitan city definition [21] dichotomised place of residence into metropolitan and rural residence. Private health medical insurance status recorded in the HMDC was used as a proxy for socio-economic status.

**Table 1:** Characteristics of the cohort (aged 25–74 years) with a first admission of ischaemic heart disease, as principal discharge diagnosis, in the period of 2002–2007, WA residents

	All			Less than 55 years			55 years and over		
	Aboriginal	Non-Aboriginal	*p*-value	Aboriginal	Non-Aboriginal	*p*-value	Aboriginal	Non-Aboriginal	*p*-value
Number of patients (%)	1269 (4.7)	25,961 (95.3)		832 (65.6)	6706 (25.8)	< 0.001	437 (34.4)	19,255 (74.2)	< 0.001
First-ever, n (%)	1041 (82.0)	22,416 (86.3)	< 0.001	700 (84.1)	6074 (90.6)	< 0.001	341 (78.0)	16,342 (84.9)	< 0.001
Sub-types of IHD, n(%)									
▪ Unstable angina	346 (27.3)	6186 (23.8)	< 0.001	224 (26.9)	1652 (24.6)	< 0.001	122 (27.9)	4534 (23.6)	< 0.001
▪ Acute myocardial infarction	456 (35.9)	7377 (28.4)		312 (37.5)	2259 (33.7)		144 (33.0)	5118 (26.6)	
▪ Other IHD	466 (36.7)	12,391 (47.7)		296 (35.6)	2793 (41.7)		170 (38.9)	9598 (49.9)	
▪ Subsequent MI	1 (0.1)	7 (0.03)		0	< 5 (0.0)		< 5 (0.2)	5 (0.0)	
Total relevant MBS records (pre-2 years)	47,047 (3.5)	1,301,191 (96.5)	< 0.001	27,805 (59.1)	228,849 (17.6)	< 0.001	19,242 (40.9)	1,072,342 (82.4)	< 0.001
1st year before index admission, n(%)	26,365 (56.0)	733,063 (56.3)	0.200	15,735 (56.6)	129,341 (56.5)	0.818	10,630 (55.2)	603,722 (56.3)	0.012
2nd year before index, n(%)	20,682 (44.0)	568,128 (43.7)		12,070 (43.4)	99,508 (43.5)		8612 (44.8)	468,620 (43.7)	
Mean age ± SD	50.2 ± 10.1	60.5 ± 9.3	< 0.001	44.4 ± 6.6	47.9 ± 5.4	< 0.001	61.4 ± 5.0	64.9 ± 5.7	< 0.001
Female sex, n(%)	574 (45.2)	7363 (28.4)	< 0.001	355 (42.7)	1621 (24.2)	< 0.001	219 (50.1)	5742 (29.8)	< 0.001
Urban/rural, n(%)									
Rural	817 (64.4)	5260 (20.3)	< 0.001	541 (65.0)	1485 (22.1)	< 0.001	276 (63.2)	3775 (19.6)	< 0.001
Urban	452 (35.6)	20,701 (79.7)		291 (35.0)	5221 (77.9)		161 (36.8)	15,480 (80.4)	
ARIA classification, n(%)									
Highly accessible	282 (22.2)	12,641 (48.7)	< 0.001	177 (21.4)	3179 (47.5)	< 0.001	103 (23.9)	9454 (49.2)	< 0.001
Accessible	236 (18.6)	9362 (36.1)		155 (18.7)	2390 (35.7)		81 (18.8)	6971 (36.3)	
Moderately accessible	202 (15.9)	2723 (10.5)		135 (16.3)	685 (10.2)		67 (15.6)	2034 (10.6)	
Remote	42 (3.3)	416 (1.6)		25 (3.0)	111 (1.7)		17 (3.9)	295 (1.5)	
Very Remote	505 (39.8)	812 (3.1)		337 (40.7)	327 (4.9)		163 (37.8)	469 (2.4)	
Indeterminate	< 5 (0.2)	7 (0.03)		0	< 5 (0.0)		0	5 (0.0)	
With private medical insurance, n (%)	34 (2.7)	11,603 (44.7)	< 0.001	21 (2.5)	2752 (41.0)	< 0.001	13 (3.0)	8851 (46.0)	< 0.001
Charlson comorbidity index (≥ 1), n %	446 (35.2)	6817 (26.3)	< 0.001	285 (34.3)	1879 (28.0)	< 0.001	161(36.8)	4938 (25.6)	< 0.001
Other comorbidities^a^, n (%)									
➢ Hypertension	680 (53.6)	11,502 (44.3)	< 0.001	402 (48.3)	2317 (34.6)	< 0.001	278 (63.6)	9185 (47.7)	< 0.001
➢ Heart failure	130 (10.2)	1390 (5.4)	< 0.001	64 (7.7)	167 (2.5)	< 0.001	66 (15.1)	1223 (6.4)	< 0.001
➢ Chronic kidney disease	167 (13.2)	789 (3.0)	< 0.001	90 (10.8)	156 (2.3)	< 0.001	77 (17.6)	633 (3.3)	< 0.001
➢ Valvular heart disease	161 (12.7)	3367 (13.0)	0.770	86 (10.3)	459 (6.8)	< 0.001	75 (17.2)	2908 (15.1)	0.235
➢ Diabetes	665 (52.4)	5217 (20.1)	< 0.001	403 (48.4)	1010 (15.1)	< 0.001	262 (60.0)	4207 (21.9)	< 0.001
➢ COPD	169 (13.3)	1266 (4.9)	< 0.001	83 (10.0)	173 (2.6)	< 0.001	86 (19.7)	1093 (5.7)	< 0.001
➢ Cancer	59 (4.7)	3607 (13.9)	< 0.001	23 (2.8)	515 (7.7)	< 0.001	36 (8.2)	3092 (16.1)	< 0.001
➢ Cerebravascular disease	53 (4.2)	808 (3.1)	0.034	29 (3.5)	71 (1.1)	< 0.001	24 (5.5)	737 (3.8)	0.074
Mortality, n (%)									
30-day mortality post-index	24 (1.9)	332 (1.3)	0.061	12 (1.4)	45 (0.7)	< 0.001	12 (2.8)	287 (1.5)	0.034
1-year mortality post-index	70 (5.5)	788 (3.0)	< 0.001	29 (3.5)	90 (1.3)	< 0.001	41 (9.4)	698 (3.6)	< 0.001

^a^Comorbidities were coded also as concurrent to index admission, which include principal diagnosis

### Statistical analyses

Baseline characteristics and crude mortality by Aboriginal status were compared using descriptive statistics. Results are provided for broad age groups < 55 and ≥ 55 years, with 55 years as the median age of the patients. Two-tailed t- or Mann-Whitney tests (for continuous variables) and the Pearson chi-squared test (for categorical variables) were used to test for significance. Negative binomial regression log-linear models were used to model count of GP consults or specialist consults as separate dependent variables in the 2 years preceding IHD admission, with adjustment for covariates at index admission. This method was selected because the distribution of our count data showed that it was over-dispersed (highly skewed) and not suitable for Poisson regression models which assume the variance is equal to the mean. Stratified analyses [by gender, broad age group, metropolitan (vs rural), incident (vs prevalent), diabetes and CKD status] were undertaken to determine the differential in subgroups and to identify where the need for intervention was most critical. STATA version 13.1 was used for analyses.

## Results

### Patient characteristics

Of 28,331 hospitalised IHD patients identified, 1101 (7.4% Aboriginal) had no MBS records and were excluded. The final cohort comprised 27,230 patients (4.7% Aboriginal; 85% with first-ever IHD admissions). Aboriginal patients were more likely to be: younger (65.6% < 55 years vs 25.8% non-Aboriginal); women (45.2% vs 28.4%); from very remote areas (39.8% vs 3.1%); to present with acute forms of IHD (acute MI or unstable angina); and have greater comorbidity burden (Table 1). Non-Aboriginal patients were more likely to have private health insurance (Table 1). Mortality following an ischaemic event was higher in Aboriginal patients in the first year after admission (5.5% vs 3.0% Non-Aboriginal) but not significantly higher 30-days following (1.9% vs 1.3% Non-Aboriginal).

### MBS expenditure and resource utilisation costs in subgroups

A total of 1,348,238 relevant MBS claims (representing $59.7 M in expenditure) were identified in the preceding 2 years, with 3.5% (47,047) attributed to Aboriginal patients, (2.7% of expenditure) (Table 2). The mean total Medicare expenditure per person was 43.3% lower in Aboriginal patients ($1271 vs $2238 non-Aboriginal). Differences in expenditure for Aboriginal and non-Aboriginal patients ranged by sub-group, from 26.9% lower in age group < 55 years, 52.6% lower in diabetic patients and 49.6% lower in those with CKD. The difference persisted when restricted to metropolitan patients. For both Aboriginal and non-Aboriginal patients, costs were significantly higher in the year closest to the index event compared to 2nd year (56.3% vs 43.7%) preceding index admission.

**Table 2:** Costs of resource utilisation in the 2 years preceding index IHD admission stratified by subgroups

	Aboriginal (1)	Non-Aboriginal (2)	Ratio (1/2)
Total healthcare expenditure on the MBS items in 2 years preceding index admission, CPI adjusted, $(%)	1,613,868 (2.7)	58,100,000 (97.3)	
Mean healthcare cost/person	1271 ± 1391	2238 ± 2319	0.57
▪ Younger age < 55	1134 ± 1360	1551 ± 1885	0.73
▪ 55 years and older	1532 ± 1412	2477 ± 2406	0.62
▪ Men	1049 ± 1195	2067 ± 2183	0.51
▪ Women	1541 ± 1556	2670 ± 2582	0.58
▪ Metro patients	1454 ± 1424	2332 ± 2382	0.62
▪ Rural patients	1170 ± 1363	1869 ± 2013	0.63
▪ Incident patients	1186 ± 1197	2099 ± 2178	0.56
▪ Diabetic patients	1461 ± 1358	3082 ± 2572	0.47
▪ Chronic kidney disease patients	1551 ± 1445	3079 ± 2667	0.50

### Medicare-derived specialty and professional consults

**Fig. 1:**
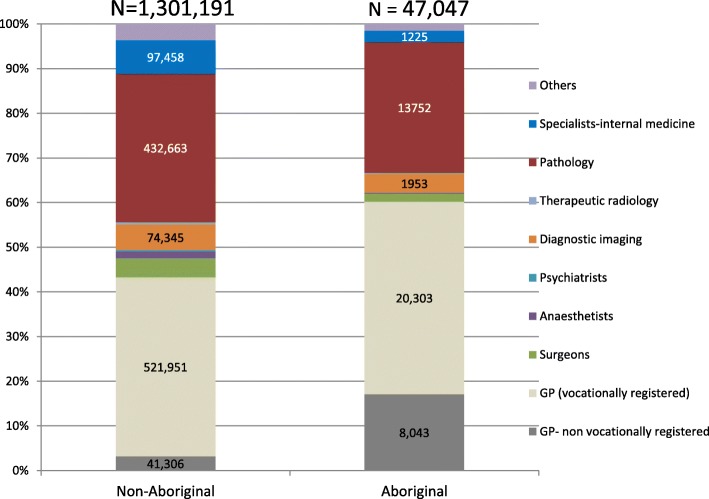
Types of different MBS health service consults and services in Aboriginal and non-Aboriginal people in the 2 years preceding the first IHD hospitalisation

Aboriginal patients had more GP records as a proportion of total records (Fig. 1) compared with non-Aboriginal patients (60.4% vs 43.8%, *p* < 0.001), although mean GP consults were not significantly different. Compared to non-Aboriginal people, Aboriginal people were 6.4 times more likely to see non-VR GPs, 69% less likely to see a specialist, 67% less likely to have surgery, 35% less likely to have diagnostic imaging and 21% less likely to have pathology tests.

Aboriginal people were more likely to have had a long/prolonged consult [defined as ‘long’: > 25 min; ‘prolonged’: > 45 min’ consultation) 16.0% vs 11.9% of total GP records]. More non-Aboriginal patients received Medicare-funded specialist consults (71.6% vs 35.0%), with a higher mean number of specialist consults (mean 4.1 vs 1.0). Mean GP costs per patient were slightly higher in Aboriginal patients ($696 vs $653) but mean Medicare-funded specialist costs were significantly lower in Aboriginal patients (Table 3). These patterns of GP and specialist consults were similar in metropolitan and rural residents.

**Table 3:** GP/specialist/allied health consultations in the 2 years preceding first IHD admission in Aboriginal versus non-Aboriginal patients

	All			Age group less than 55 years		
	Aboriginal	Non-Aboriginal	*p*-value	Aboriginal	Non-Aboriginal	*p*-value
1. Total MBS items, n (%)	47,047 (3.5)	1,301,191 (96.5)		27,805 (59.1)	228,849 (17.6)	
▪ Professional consults of total items, n (%)	30,926 (65.7)	773,278 (59.4)	< 0.001	18,482 (66.5)	133,607 (58.4)	< 0.001
▪ Non-professional consults of total items, n(%)	16,121 (34.3)	527,913 (40.6)		9323 (33.5)	95,242 (41.6)	
2. Types of consults or services of total items, n (%)						
▪ Total GP records (of total MBS items)	28,392 (60.4)	569,763 (43.8)	< 0.001	17,222 (61.9)	100,581 (44.0)	< 0.001
▪ Total specialist records (of total MBS items)	2443 (5.2)	204,002 (15.7)		1207 (4.3)	33,142 (14.5)	
▪ Allied health services (of total MBS records)	134 (0.3)	4074 (0.3)		68 (0.2)	740 (0.3)	
▪ Other MBS services -pathology	14,127 (30.0)	439,202 (33.7)		8174 (29.4)	78,754 (34.4)	
▪ Other services	1951 (4.1)	84,150 (6.5)		1134 (4.2)	15,632 (6.8)	
Mean # MBS items per day per patient (note pts. can have two consults/day)	1.8 ± 1.2	2.0 ± 1.6	< 0.001	1.8 ± 1.2	1.9 ± 1.6	< 0.001
3. Types of professional consults, n(%)	30,926 (65.7)	773,278 (59.4)	< 0.001	18,482 (66.5)	133,607 (58.4)	< 0.001
▪ Number of GP consults	28,392 (91.8)	569,763 (73.7)	< 0.001	17,222 (93.2)	100,581 (75.3)	< 0.001
▪ Number of specialist consults	2443 (7.9)	204,002 (26.4)	< 0.001	1207 (6.5)	33,142 (24.8)	< 0.001
▪ Number allied health consults	134 (0.4)	4074 (0.5)	0.025	68 (0.4)	740 (0.5)	0.001
Sub-total of professional consults	30,926	773,278	< 0.001	18,482	133,607	< 0.001
GP consult category, n (%)						
▪ GP consult (short-prolonged) category unspecified	8319 (29.3)	160,511 (28.2)	< 0.001	5063 (29.4)	19,926 (19.8)	< 0.001
▪ Short	492 (1.7)	6185 (1.1)		280 (1.6)	1048 (1.0)	
▪ Standard	15,039 (53.0)	336,162 (59.0)		9104 (52.9)	65,965 (65.6)	
▪ Long	4087 (14.4)	61,871 (10.9)		2505 (14.6)	12,498 (12.4)	
▪ Prolonged	455 (1.6)	5034 (0.9)		270 (1.6)	1144 (1.1)	
4. Patient counts						
Patients with no MBS records), n (%)	102 (7.4)	999 (3.7)	0.035	80 (5.8)	410 (1.5)	0.008
Number of patients with MBS records, n (%)	1269 (92.5)	25,961 (95.3)		832 (60.6)	6706 (24.6)	
5. GP consults, person-based						
Patients with no GP consults, n (%)	75 (5.9)	590 (2.3)	< 0.001	52 (6.3)	146 (2.2)	< 0.001
Patients with ≥1 GP consults, n (%)	1194 (94.1)	25,371 (97.7)		780 (93.7)	6560 (97.8)	
Mean GP visits-person-based, ±SD	19.1 ± 32.6	13.9 ± 15.1	< 0.001	17.2 ± 25.3	13.6 ± 14.4	< 0.001
Mean GP costs/person, dollars ±SD	696.15 ± 780.9	653.72 ± 584.9	0.007	668 ± 908.7	507.9 ± 543.7	< 0.001
Specialist consults, person-based						
Patients with no specialist consults, n (%)	825 (65.0)	7376 (28.4)	< 0.001	587 (70.6)	2799 (41.7)	< 0.001
Patients with specialist consults, n (%)	444 (35.0)	18,585 (71.6)		245 (29.4)	3907 (58.3)	
Mean specialists visits, person-based	1.7 ± 4.1	7.6 ± 14.4	< 0.001	0.7 ± 1.6	2.6 ± 5.9	< 0.001
Mean specialist costs/person, dollars±SD	662.5 ± 808.7	1365.26 ± 1658.3	< 0.001	642.0 ± 817.2	1060.1 ± 1480.4	0.009

More non-Aboriginal (vs Aboriginal) patients consulted a GP in the 30 days before first IHD admission (12.6% vs 10.9%, *p* < 0.001). In the year preceding first IHD admission, the median time between last GP visit and index admission was slightly (but significantly) longer for Aboriginal patients [161 (IQR 74–264) vs 159 (IQR 69–260)] days. Specific MBS items for Aboriginal and telehealth services were examined but the counts were relatively low, as the period (2000–2007) pre-dated Medicare funding for telehealth.

### ED presentations

59.3% of non-Aboriginal patients (vs 86.4% Aboriginal) had prior ED records. Of 33,170 ED attendances in the 2 years preceding IHD, 14% were attributed to Aboriginal patients. Aboriginal (vs non-Aboriginal) patients had higher mean ED visits (4.3 vs 2.6), *p* < 0.001. When restricted to urgent/semi-urgent attendances, mean number of ED presentations was higher in Aboriginal people (2.9 vs 1.9). Furthermore, the mean interval from the last urgent ED presentation to index admission was shorter in Aboriginal patients (27.9 vs 37.4 days, *p* < 0.001), respectively.

### Independent predictors of GP and specialist consults

Increasing age, female sex and comorbidities were all independently associated with higher rates of GP and specialist consults (Table 4). Heart failure and cerebrovascular disease, however, were associated with increased rates of GP but not Medicare-funded specialist consults. Private medical insurance was the strongest independent predictor of increased specialist service usage. Notably, there was a declining gradient of GP and specialist consultations as residential postcodes became more remote (Table 4). In the fully adjusted model, Aboriginal status was associated with a slightly higher IRR of 1.07 (95% CI 1.02–1.12) for GP consults but an IRR of 0.44 (95% CI 0.40–0.48) for Medicare-rebated specialist consults. The latter IRR reduced further to 0.36 when the analysis was restricted to only metropolitan patients.

**Table 4:** Multivariable models using negative binomial regression for rates of GP visits (truncated at max 104, allowing maximum of 1 GP visit per week) and specialist consults over 2 years prior to first IHD admission (*n* = 27,230)

Multivariable model for GP consults					Multivariable model for specialist consults			
GP visits (truncated)	IRR	*p*-value	95% CI		IRR	*p*-value	95% CI	
Age at admission	1.02	< 0.001	1.02	1.02	1.03	< 0.001	1.02	1.03
Female gender	1.32	< 0.001	1.30	1.35	1.32	< 0.001	1.28	1.37
Year of admission	0.96	< 0.001	0.95	0.96	0.95	< 0.001	0.94	0.96
Aboriginal status (1)	1.07	0.004	1.02	1.12	0.44	< 0.001	0.40	0.48
With private medical insurance	0.90	< 0.001	0.88	0.92	1.81	< 0.001	1.75	1.86
ARIA classification								
Highly accessible	1.00				1.00			
Accessible	0.93	< 0.001	0.91	0.94	0.92	< 0.001	0.89	0.96
Moderately accessible	0.82	< 0.001	0.79	0.84	0.80	< 0.001	0.76	0.84
Remote	0.86	< 0.001	0.80	0.92	0.82	0.002	0.72	0.93
Very remote	0.66	< 0.001	0.63	0.69	0.44	< 0.001	0.40	0.48
Comorbidities								
Heart failure	1.04	0.031	1.00	1.09	1.02	0.629	0.95	1.09
Chronic kidney disease	1.19	< 0.001	1.13	1.25	1.53	< 0.001	1.40	1.66
Hypertension	1.17	< 0.001	1.15	1.19	1.07	< 0.001	1.03	1.10
Rheumatic/valvular heart disease	1.14	< 0.001	1.11	1.17	1.25	< 0.001	1.20	1.31
Diabetes	1.33	< 0.001	1.30	1.36	1.47	< 0.001	1.41	1.53
COPD	1.42	< 0.001	1.37	1.48	1.42	< 0.001	1.33	1.52
Cancer	1.2	< 0.001	1.17	1.23	1.71	< 0.001	1.63	1.78
Cerebrovascular disease	1.29	0.002	1.10	1.51	1.23	0.145	0.93	1.63
Stroke	0.98	0.835	0.83	1.16	1.04	0.817	0.77	1.39
Coronary heart disease	0.77	< 0.001	0.70	0.83	0.71	< 0.001	0.61	0.82
Charlson index	0.89	< 0.001	0.88	0.9	0.88	< 0.001	0.86	0.89

IRR = incidence rate ratio; 95% CI = 95% confidence interval

Notes: For age group under 55 years and separately, in patients 55 years and older, there was no significant difference between Aboriginal and non-Aboriginal patients for GP visits (truncated at 104). When restricted to only metropolitan patients, Aboriginal patients had an adjusted IRR of only 0.36 (95% CI 0.31–0.42, *p* < 0.001) for specialist visits (as a count variable). Having private medical insurance was negatively associated with GP visits but positively with specialist visits, the IRR is 1.84 (95% CI 1.77–1.90) compared with those without private medical insurance

## Discussion

This person-level, population-based study investigated the patterns of Medicare-funded GP and specialist care, and resource utilisation for Aboriginal and non-Aboriginal patients in the 2 years preceding first IHD hospitalisation. Our findings show that, after adjustment, Aboriginal patients were more likely to have had a GP consultation, a long/ prolonged consult and consult a non-VR GP, while substantially fewer received Medicare-rebated specialist consultations. The mean cost differential on Medicare expenditure over 2 years was 43.3% lower in Aboriginal compared to non-Aboriginal people, despite higher prevalence of multimorbidity in Aboriginal patients. This difference persisted in metropolitan subgroups. Notably, the resource differential was greatest in diabetic and CKD patients for whom ongoing specialist expertise for managing complex care needs is arguably more critical. Private specialist consults were the key cost driver in Medicare expenditure differentials between Aboriginal and non-Aboriginal people. For ED presentations, Aboriginal patients, particularly those in rural/remote areas were more likely to use ED than GPs for conditions classified urgent/semi-urgent. These findings suggest a substitution effect of ED with primary care.

Published empirical evidence demonstrates the association between a strong primary care system and better health status, reduced hospitalisation, and all-cause (and CVD) mortality [22–24]. Further, evolving research suggests that a collaborative multidisciplinary approach based on the integration of specialists and GPs could lead to better cardiovascular outcomes in primary care setting, particularly for patients with multimorbidity [25]. In our study there was an over-representation of Aboriginal patients with no primary care records in the pre-event study period. Additionally, although Aboriginal patients had a marginally higher adjusted-IRR for primary consults, over a quarter of GP consults were rendered by non-VR GPs/IMGs. This might impact on the continuity and cultural appropriateness of care. IMGs are strongly represented in rural/remote Australia (particularly WA) [12] with many relocating from rural areas once licensing restrictions are satisfied [12, 26]. In a study of IMGs, Durey et al. [27] identified the need to better address cross-cultural issues and the importance of effective communication and building community and cross-institutional relationships. A strong physician-patient relationship (particularly developed in a culturally secure context), is a key element enabling continuity of care necessary for optimal management of chronic diseases, and might be limited in the current environment.

High performing general practices have lower IHD admission and mortality rates, with the association strongest for practices serving populations of high levels of economic deprivation [28]. This may lead to a reduction in the health inequalities noted in this analysis. Despite the initiatives to strengthen the GP sector in very remote areas, poor access to GP and both Medicare-funded and public hospital specialist services persists in very remote areas where the majority of Aboriginal people live. This highlights a critical need to create more innovative models of care.

Aboriginal patients, despite their younger average age, have significantly greater co-morbidity, adding considerably to complexities in managing the primary disease. This is often accompanied by complex social circumstances resulting from the unfortunate historical and political legacy of colonialism, with adversities including living in under-resourced remote locations (where access to medical care may be limited), poverty, unhealthy environments and higher rates of mental illness, imprisonment, poor educational attainment and family troubles [29]. This social, clinical and logistical complexity may explain the higher proportion of long/prolonged visits. Higher ED use among Aboriginal patients may reflect poor access to and/or out-of-pocket costs of primary care (where ED is used as a proxy for primary care) as well as high clinical need. Comorbidities were all independent predictors of increased rates of GP consultations (Table 4). Consequently, Aboriginal (vs non-Aboriginal) patients have poorer 30-day and 1-year mortality outcomes, consistent with other reports [16, 17].

Rural disparities in Medicare expenditure for the financial year 2006–2007 were reflected in a total Medicare deficit of $811 million [30], translating into 12.6 million fewer services during that year for the people of regional and remote areas, attributed largely to poorer access to health professionals. We have shown similar under-expenditure across the broad MBS service categories among Aboriginal patients, particularly in poorer access to specialist care. As Medicare-rebated specialist care does not cover public hospital out-patient services, the exact quantification of differential specialist utilisation could not be captured. Nevertheless, access to specialist care remains a significant issue for Aboriginal cardiac patients, often reflecting specialist shortages in rural areas, under-developed care pathways and weaknesses in communication/information flow between primary and acute and specialist care professionals – all of which impact on quality of care. Since 2010, Medicare has introduced a wide range for MBS items and rebates, including those for telehealth consultations for medical specialists in other locations, as a means to improve access. It is possible that greater uptake of such initiatives could address some of this gap.

More innovative models of care, incorporating appropriate telehealth, are needed to overcome the problems of costs and accessibility related to geographical location in WA. Additionally, this analysis shows structural reform of PHC services is needed to ensure better integration of care and management for Aboriginal people, over half of whom reside in rural Australia. Analysis of more contemporary data will be valuable to show how service utilisation has changed with recent health reforms.

### Strengths and limitations

This study uses MBS data linked to a diagnostic cohort identified through hospital data, allowing individualised *person-based* utilisation of specific services and associated costs to be examined. Medicare data represent the most accurate and reliable source of health care attendance in Australia, although visits to an Aboriginal Medical Service could not be differentiated in provided data from mainstream consults. MBS data do not include outpatient consultations at public hospitals and the use of MBS data alone limits the true representation of specialist services provided. As Aboriginal patients are over-represented in the (mainly metropolitan) public outpatient clinics in WA and some specialist visits occur through alternative funding mechanisms, the difference in receipt of specialist ambulatory consults may be over-estimated. Improvement of out-patient hospital data to linkage standard is urgently required. Further, the MBS data is relatively old due to the long lead time needed for the requisition of Commonwealth data; regardless, the current analysis provides a base from which to gauge further improvements with ‘Closing the Gap’ initiatives undertaken by the government.

## Conclusions

Despite being over-represented in admissions for IHD, Aboriginal people were under-resourced compared with the rest of the population, particularly in terms of Medicare-funded specialist care. The differential in specialist consultations was the main driver of the difference in Medicare expenditure between the sub-populations. The disparity in resource utilisation is most marked among Aboriginal patients with diabetes and CKD, major contributors to premature illness and health costs. The findings underscore the need to further strengthen the continuity and integration of care (in and between primary and specialist care) for remote/very remote areas and for innovations in service delivery models involving shared primary and specialist care.

## Abbreviations

ARIA: Accessibility/Remoteness Index of Australia
CKD: Chronic kidney disease
CVD: Cardiovascular disease
ED: Emergency departments
EDDC: Emergency Department Data Collection
GP: General practitioners
HMDC: Hospital morbidity data collection
IHD: Ischaemic heart disease
IMGs: International medical graduates
MBS: Medicare benefits schedule
VR GPs: Vocationally registered general practitioners
WA: Western Australia
